# Call for Decision Support for High-Alert Medication Administration Among Pediatric Nurses: Findings From a Large, Multicenter, Cross-Sectional Survey in China

**DOI:** 10.3389/fphar.2022.860438

**Published:** 2022-07-19

**Authors:** Mengxue He, Qin Huang, Hong Lu, Ying Gu, Yan Hu, Xiaobo Zhang

**Affiliations:** ^1^ School of Nursing, Fudan University, Shanghai, China; ^2^ Fudan University Centre for Evidence-based Nursing: A Joanna Briggs Institute Centre of Excellence, Fudan University, Shanghai, China; ^3^ Shanghai Children’s Medical Center, Affiliated to Shanghai Jiaotong University, School of Medicine, Shanghai, China; ^4^ Children's Hospital, Fudan University, Shanghai, China

**Keywords:** high-alert medications, knowledge, nurses, pediatrics, medication errors, decision support

## Abstract

**Background:** Children have a higher risk of medication errors (MEs) than adults. The Institute for Safe Medication Practice (ISMP) defined high-alert medications (HAMs) as a group of medications that could cause significant patient harm or even death when they are used in error. Nurses are actively involved in and responsible for patient care, especially in medication administration. This study aimed to estimate the knowledge, decision-making basis and confidence and decision support needs related to HAMs among pediatric nurses in China.

**Methods:** A web-based, cross-sectional survey was conducted among pediatric nurses who were recruited from 14 member hospitals of the Pediatric Nursing Alliance of National Children’s Medical Center in China using a convenient sampling technique. Data were collected using a self-administered instrument composed of four parts: the demographic characteristics of participants, participants’ knowledge about HAMs, participants’ self-evaluation of the basis of and confidence in decision-making, and decision support needs regarding HAMs. Among the participants, the maximum score for HAM knowledge was 100. All data were entered and analyzed using SPSS 20.0.

**Results:** A total of 966 nurses participated in this study. Nurses were found to have insufficient knowledge about HAMs, with a median (IQR) of 75.0 (70.0, 80.0), out of a maximum score of 100. Knowledge about HAM administration was significantly higher than that about HAM regulation, with a *p* value < 0.001. The three lowest-scoring items concerned HAM regulation, and the “Treat fentanyl skin patches as a regulated narcotic” item obtained the lowest score, with only 1/5 of respondents answering it correctly. Most participants reported that their basis for decision-making about HAMs was drug instructions (90.0%) or drug handbooks (81.9%) and evaluated their confidence in decision-making about HAMs as high or relatively high (84.6%). The decision-making difficulties when encountering HAMs focused on most stages of HAM administration, especially the appropriateness of prescriptions, checks, preparation and administration. The vast majority of participants assessed decision support as necessary or very necessary (92.0%), and the most popular options for decision support were computerized clinical decision support systems (46.4%) and real-time online communication with pharmacists (23.9%).

**Conclusion:** Our study demonstrated the inadequacies in HAM knowledge, the basis and difficulty of decision-making, and decision support needs regarding HAMs in Chinese pediatric nurses. Nurses need greater support in HAM administration, including not only training but also adequate technology, mutually beneficial interprofessional collaboration, and a positive institutional culture.

## Introduction

According to the National Coordinating Council for Medication Error Reporting and Prevention (NCCMERP), medication error (ME) is defined as any preventable event that may cause or lead to inappropriate medication use or patient harm while the medication is in the control of the health care professional, patient or consumer (National Coordinating Council for Medication Error Reporting and Prevention, 2021). Due to their unique developmental, cognitive, medical, and pharmacokinetic characteristics, pediatric patients have a higher risk of MEs than adult patients (Grissinger, 2015). Hospitalized children may experience three times as many MEs as hospitalized adults, and these errors are frequently harmful (Kaushal et al., 2001). Such events may occur at any stage of the medication process, including prescribing, transcribing/verifying, packaging, compounding, dispensing/delivering, distribution, administration, and monitoring (Gonzales, 2010). Nurses are actively involved in patient care, especially in medication administration; medication administration is the last opportunity to correct an error before a potential ME actually occurs for a patient. As the most extensive members of the healthcare system, nurses often act as a key source of system-level resilience in improving medication safety owing to their close contact with patients throughout healthcare service (Vos et al., 2020).

High-alert medications (HAMs) are a group of specific medications that can cause significant patient harm or even death when they are used in error; this classification was proposed by the Institute for Safe Medication Practice (ISMP) (Institute for Safe Medication Practices, 2018). Compared to ordinary drugs, HAMs bear greater risks of adverse events. Through analysis of all pediatric drug-related incident reports to the Health and Social Care Inspectorate in Sweden 2011–2017, Nydert et al. (2020) found that the prevalence of incident reports involving HAMs was almost double that of nonalert substances. The ISMP developed the first list of HAMs in acute care settings in 1995, and the newest version of the list was published in 2018 (Institute for Safe Medication Practices, 2018). Several countries established their own lists of HAMs based on the ISMP list (Khoo et al., 2013; Maaskant et al., 2013; Hospital Pharmacy Committee of Chinese Pharmaceutical Assiciation, 2019), and most lists include the following classes: chemotherapeutic agents, highly concentrated electrolytes, neuromuscular blocking agents, cardiovascular medications, anticoagulants, opioids and their derivatives, and benzodiazepines. Some HAMs have a narrow therapeutic range, e.g., chemotherapeutic agents, which, when used at improper doses, may cause undesirable events; other HAMs require special administration methods, e.g., vincristine is for intravenous use only, vasoactive agents have high-level requirements for intravenous catheters, and parenteral nutrition requires a strict administration rate and monitoring of side effects. Correct and appropriate administration of HAMs is a major concern for nurses worldwide and is directly related to the safety and quality of patient care.

Insufficient knowledge among nurses is often considered to be a major factor in MEs (Tang et al., 2007), and several studies of knowledge about HAMs have been conducted among nurses (Apolinário et al., 2019; Zyoud et al., 2019). Inadequate training for nurses, negative influences from other professionals, and experiences of incorrect practice in administration indeed jeopardize medication safety. However, knowledge lays the foundation of clinical practice, and decision-making based on reasoning in the clinical context is essential for medication safety (Dickson and Flynn, 2012). According to Sulosaari et al. (2011), nurses’ medication competence consists of three major areas: theoretical, practical and decision-making competence. Ideally, clinical decisions should be made based on the best available evidence, but studies have shown that this is not always true (Goethals et al., 2012; Kozlowski et al., 2017). The traditional dual process theories emerging from the basic psychological sciences propose two kinds of thinking processes, including system 1, which is automatically activated by environmental cues, and system 2, which involves deeper processing and conscious reasoning (Weir et al., 2017). Given that decision-making is a cognitive process involving the available alternatives to solve clinical problems, it is undoubtedly affected by personal knowledge, experience and environmental factors (Choi and Kim, 2015). To the best of our knowledge, there has been no study investigating the basis of decision-making about HAM administration, both in pediatric nurses and other specialties. In addition, published data are also not available about the needs of decision support about HAM administration. Therefore, this study aims to assess the knowledge, basis of and confidence in decision-making and decision support needs regarding HAMs among Chinese pediatric nurses.

## Materials and Methods

Due to the regional lockdown and social distancing rules enforced during the COVID-19 pandemic, a web-based, cross-sectional, descriptive study was conducted to explore the knowledge, confidence in decision-making, and decision support needs regarding HAM administration among Chinese pediatric nurses.

### Setting and Participants

The Pediatric Nursing Alliance of National Children’s Medical Center supported this study. Participants in this study were pediatric nurses working in the 14 tertiary Children’s Hospital members of the abovementioned alliance, including 5 in East China, 4 in South and Central China, 2 in North China, 1 in Northeast China, 1 in Southwest China, and 1 in Northwest China. The inclusion criteria were as follows: 1) pediatric nurses working in wards and ICUs; 2) nurses working for at least 1 year; and 3) nurses who were fluent in Chinese, understood the research objectives and content of the questionnaires, and gave informed consent. Nursing students were excluded from this study.

The sample size needed for this study was calculated using the Raosoft sample size calculator to achieve a confidence level of 95%, a margin of error of 5%, and a response distribution of 50% (Raosoft Inc, 2021). Among these, the confidence level is the tolerable amount of uncertainty, and the margin of error is the tolerable amount of error. A minimum of 377 nurses was required for this investigation. However, with respect to the limitations of convenience sampling and a web-based survey design, we empowered the sample size by including a design effect of 2 for a convenience-sampled study, which made the adjusted minimum sample 754 (Masoud et al., 2021).

### Ethical Approval

The protocol of this study was reviewed and approved by the Research Ethics Committee of the Children’s Hospital of Fudan University [No. (2021)333]. All the participants were ensured of the confidentiality of the investigation.

### Study Instrument

Demographic information was collected, including age (years), sex (male/female), parental status, pediatric nursing experience (years), educational level, seniority status, working department, and position. There were several items regarding the Chinese nurses’ background information that are worth mentioning. There were four levels of nursing education in China: an associate degree (3–4 years of occupational education), a bachelor’s degree (4–5 years of an undergraduate program), a master’s degree and a doctoral degree. With respect to position, the primary type of nurses were primary nurses or bedside nurses. Regarding seniority, there were 4 professional levels of nursing staff: a junior registered nurse (RN), a senior RN, a nurse in charge, and a professor nurse. While the nurses might work in different departments in succession, they were required to report their predominant one Department. The present training, clinical practice, and error experience in HAM administration were also investigated, and the error experience included both the nurses’ own errors or errors they witnessed and both reported and not reported errors.

A questionnaire developed by Hsaio et al. (2010) was used to examine knowledge of HAMs. This instrument has two parts, each with 10 items assessing HAM administration and regulation. Each correct answer received 1 point, while wrong or “don’t know” answers received 0 points. Therefore, the total possible score for HAM-related knowledge was 20 points, and the total score was 100 points after recalculation. Knowledge scores were classified as good knowledge ≥70%, or poor knowledge <70%, as in previous study (Salman et al., 2020). The original instrument was in English. Permission to use this tool and translate it into simplified Chinese was obtained from the corresponding author. We did a standard “forward-backward” procedure (Wild et al., 2005). Three experts in the fields of pediatrics, pediatric nursing and nursing education tested the content validity index (CVI) of this questionnaire and found out the scale-level CVI score was 0.97 and the item-level CVI score was 0.67–1. Then, a preliminary test of the Chinese version was conducted with 20 nurses. All participants reported that the questionnaire was clear and easy to understand. The Cronbach’s alpha of this questionnaire was 0.830, showing adequate internal consistency, similar to a previous study (Mustafa et al., 2022). The results from the pilot test were not included in the final analysis of the data.

Then, a self-evaluation section was designed to assess nurses’ decision-making and decision-support about HAMs; it included 5 parts: ① basis for decision-making when administrating HAMs, which included 12 multiple-select multiple-choice items; ② confidence in decision-making when administrating HAMs, including four levels (‘high’, ‘relatively high’, ‘relatively low’, and ‘low’); ③ difficulties in decision-making when administrating HAMs, including 7 multiple-choice items; ④ needs for decision support about HAMs, including 4 response options (“very necessary”, “necessary”, “uncertain”, and “not necessary”); and ⑤ preferred forms of decision support about HAMs, including five multiple-choice items. The three above-mentioned experts also tested the CVI of this self-evaluation section; the results showed that the scale-level CVI score was 0.85. The final questionnaire is shown in the Supplementary Material.

### Data Collection

We used the Wenjuanxing website (https://www.wjx.cn/), which is a secure web-based platform designed for capturing data from online investigations. This platform was used to create a survey project located on a static URL that could be remotely accessed via a smartphone browser, regardless of the geographical position of the proxy. There was a web-based informed consent page for obtaining e-Consent prior to the online study questionnaire page, and consenting questions were added to the page with a yes/no format. During the enrollment process, if a participant provided an answer that disagreed with a consenting statement, the platform introduced a hard stop feature, preventing ineligible participants from enrolling in the study.

The research team recruited from hospitals that gave their permission after reviewing the research protocol and the IRB approval. Then, data were collected from August 1, 2021, to September 23, 2021. A convenient sampling technique was employed. To control for possible confounding variables, we asked the nursing departments to choose a minimum of 3 units in each hospital. Then, the lists of nurses in these units were provided by the nursing department as a sampling frame. Trained investigators approached the nursing staff in the above-mentioned units and briefed them about the intent of this study; then, those who were willing to be enrolled received the web-based anonymous questionnaire. Every participant was informed once. Multiple entries from the same individual were prevented by the unique IP address according to the platform setting.

### Data Analysis

Only complete questionnaires were included in the final analysis. Data were analyzed using SPSS 20.0 (SPSS, Inc., Chicago, IL. United States). Categorical data, including most demographic data and the basis of and confidence in HAM decision-making, are presented as numbers, percentages and frequencies. Continuous variables, including age, pediatric nursing experience, and knowledge about HAMs, were tested for normality by the Kolmogorov-Smirnov test and presented as the mean ± SD or median with lower-upper quartiles, as appropriate. Then, categorical data were compared with the chi-square test. Continuous data were compared between groups by the *t*-test or Mann-Whitney *U* test, as applicable. A *p* value of less than 0.05 was considered statistically significant.

## Results

A total convenience sample of 2,518 nurses were approached, and 1,033 nurses took part in this survey, with a response rate of 41.0%. After excluding 67 questionnaires with missing data, the completion rate was 93.5%; ultimately, 966 complete questionnaires were obtained. The flowchart of participant recruitment is shown in the Supplementary Material.

### Demographic Characteristics

Table 1 shows the demographic characteristics of the study participants. The majority of participating nurses were female (97.5%). The median (IQR) age and pediatric nursing experience were 31.0 (27.0, 35.0) years and 8.0 (4.0, 13.0) years, respectively. A total of 336 participants (34.8%) worked in ICUs, including neonatal ICUs and other regular pediatric ICUs. The rest of the participants (65.2%) worked in pediatric wards.

**TABLE 1:** Demographic characteristics (*N* = 966).

Variables	n (%)	M(P25, P75)
Age (years)	—	31.0 (27.0, 35.0)
Female sex	942 (97.5)	—
Pediatric nursing experience (years)	—	8.0 (4.0, 13.0)
Working departments		
ICU	336 (34.8)	—
Non-ICU	630 (65.2)	—
Positions		
Assistant nurse	13 (1.3)	—
Primary nurse	708 (73.3)	—
Head nurse	72 (7.5)	—
Nurse educator/specialized nurseetc.	173 (17.9)	—
Educational level		
Associate degree	573 (59.3)	—
Bachelor’s degree	378 (39.1)	—
Master’s degree or higher	15 (1.5)	—
Seniority		
Junior	644 (66.7)	—
Intermediate	286 (29.6)	—
Senior	36 (3.7)	—
With children	569 (58.9)	—
Frequency of HAM administration		
≥1 per day	208 (21.5)	—
≥1 per week	299 (31.0)	—
≥1 per month	247 (25.6)	—
≥1 per year	212 (21.9)	—
Training about HAMs		
In orientation training	174 (18.0)	—
In in-service training	228 (23.6)	—
In both orientation and in-service training	536 (55.5)	—
None	28 (2.9)	—
Experience of medication administration error	323 (33.4)	—
Involved with HAMs	150 (15.5)	—
All reported instances of HAM administration error	115 (11.9)	—
All report instances of non-HAM administration error	147 (15.2)	—

### Knowledge About HAMs

Out of a maximum score of 100, the study participants’ total score of knowledge about HAMs was 75.0 (70.0, 80.0). A paired Wilcoxon test (Z = -5.293, *p* < 0.001) showed that knowledge about HAM administration [40.0 (35.0, 45.0)] was significantly higher than knowledge about HAM regulation [35.0 (35.0.40.0)]. A total of 228 (23.6%) of the study participants had scores <70. The three highest-scoring items for both groups were as follows: “Distinctive labeling should be used on look-like drugs” (97.5%); “If a ward stores atracurium for tracheal intubation, the drug should be stored with other drugs and easily accessed by nurses” (96.2%); and “When an emergency such as ventricular fibrillation happens, push fast 15% potassium chloride (KCL) 10 ml intravenously” (96.9%). The three lowest-scoring questions for both groups all concerned HAM regulation: “Treat fentanyl skin patches as a regulated narcotic” (21.1%); “Each drug should have multiple concentrations for the nurse to choose from” (34.3%); and “Use “U” instead of unit for dose expression” (39.6%). When the participant sample was divided based on a cut-off score of 70, the two groups differed in age, and pediatric nursing experience, as shown in Table 2.

**TABLE 2:** Knowledge about HAMs (*N* = 966).

Variables		Total Score ≥70	Total Score <70	Z	X^2^	*P*
Age [M(P25, P75), years]	—	31.0 (27.0, 36.0)	30.0 (27.0, 33.0)	−3.143^#^		0.002^^^
Pediatric nursing experience [M(P25, P75), years]	—	9.0 (5.0, 14.0)	7.0 (4.0, 10.25)	−3.757^#^		0.000^^^
Working department [n(%)]	ICU	265(78.9)	71(21.1)	—	1.745^&^	0.107
	Non-ICU	473(75.1)	157(24.9)	—	—	—
Primary nurse [n(%)]	Yes	531(75.0)	177(25.0)	—	2.871^&^	0.052
	No	207(80.2)	51(19.8)	—	—	—
Sex [n(%)]	Male	722(76.6)	220(23.4)	—	1.292^&^	0.183
	Female	16(66.7)	8(33.3)	—	—	—
With children [n(%)]	Yes	444(78.0)	125(22.0)	—	2.050^&^	0.088
	No	294(74.1)	103(27.9)	—	—	—
Bachelor’s degree or higher level of education [n(%)]	Yes	299(82.4)	94(17.6)	—	0.037^&^	0.453
	No	439(76.6)	134(23.4)	—	—	—
Intermediate or higher level of seniority [n(%)]	Yes	262(81.4)	60(18.6)	—	6.614^&^	0.006^^^
	No	476(73.9)	168(26.1)	—	—	—
Training about HAMs [n(%)]	Yes	717(76.4)	221(23.6)	—	0.031^&^	0.504
	No	21(75.0)	7(25.0)	—	—	—

^#^Mann-Whitney *U* test;^&^Chi square test, ^^^ *p* < 0.01. *p < 0.05.

### Basis of and Confidence in Decision-Making About HAMs

Regarding the basis of decision-making about HAMs, the most frequent choice was drug instructions (878, 90.0%), followed by drug handbooks (791, 81.9%), doctors (750, 77.6%), pharmacists (561, 58.1%), clinical practice guidelines (527, 54.6%), textbooks (481, 49.8%), colleagues (404, 41.8%), personal experiences (391, 40.5%), supervisors (349, 36.1%), ideas from academic conferences (171, 17.7%), systematic reviews (169, 17.5%), and original studies (85, 8.8%).

With regard to confidence in decision-making about HAMs, 114 nurses (11.8%) reported high-level confidence, 704 (72.9%) reported relatively high-level confidence, 130 (13.5%) reported relatively low-level confidence, and 18 (1.9%) reported low-level confidence. When those with relatively high-level and high-level confidence were grouped as having “high-level confidence” and those with relatively low-level confidence and low-level confidence were grouped as having “low-level confidence”, the two groups showed different characteristics, as shown in Table 3.

**TABLE 3:** Level of confidence of decision-making (*N* = 966).

Variables		High-level Confidence	Low-level Confidence	Z	X^2^	*P*
Age [M(P25, P75), years]	—	31.0 (28.0, 36.0)	30.0 (25.75, 32.0)	−4.768^#^	—	0.000^^^
Pediatric nursing experience [M(P25, P75), years]	—	9.0 (5.0, 13.0)	6.0 (3.0, 10.0)	−5.060^#^	—	0.000^^^
HAM knowledge score less than 70 [n(%)]	Yes	178(78.1)	50(21.9)	—	10.047^&^	0.001^^^
	No	640(86.7)	98(13.3)	—	—	—
Working department [n(%)]	ICU	293(87.2)	43(12.8)	—	2.528^&^	0.066
	Non-ICU	525(83.3)	105(16.7)	—	—	—
Primary nurse [n(%)]	Yes	590(83.3)	118(16.7)	—	3.701^&^	0.032^^^
	No	228(88.4)	30(11.6)	—	—	—
Sex [n(%)]	Male	22(91.7)	2(8.3)	—	0.926^&^	0.262
	Female	796(84.5)	146(15.5)	—	—	—
With children [n(%)]	Yes	495(87.0)	74(13.0)	—	5.722^&^	0.011*
	No	323(81.4)	74(18.6)	—	—	—
Bachelor’s degree or higher level of education [n(%)]	Yes	336(85.5)	57(14.5)	—	0.341^&^	0.312
	No	482(84.1)	91(15.9)	—	—	—
Intermediate or higher level of seniority [n(%)]	Yes	289(89.8)	33(10.2)	—	9.579^&^	0.001^^^
	No	529(82.1)	115(17.9)	—	—	—
Training about HAMs [n(%)]	Yes	799(85.2)	139(14.8)	—	6.290^&^	0.019*
	No	19(67.9)	9(32.1)	—	—	—

^#^Mann-Whitney *U* test. ^&^Chi square test, ^^^ *p* < 0.01, *p < 0.05.

### Difficulty of Decision-Making and Needs for Decision-Support About HAMs

In regard to the difficulties of decision-making about HAMs, the results showed that the decision-making regarding the appropriateness of prescriptions was the most difficult [7.0(5.0.8.0)], followed by “Three Checks Seven Rights” (the typical common rule for Chinese nurses to check that the patient and medication are correct) [7.0(4.0,7.0)], medication preparations [6.0(4.0,6.0)], medication administration [5.0(4.0,6.0)], effectiveness evaluations [4.0(3.0,6.0)], monitoring adverse reactions [4.0(3.0,7.0)], and providing health instructions about drugs for patients and their parents [3.0(2.0,4.0)].

Regarding the needs for decision support about HAMs, 461 nurses (47.7%) considered decision support “very necessary”, 428 (44.3%) considered it “necessary”, 11 (1.1%) were “uncertain”, and 66 (6.8%) considered it “not necessary”. When “very necessary” and “necessary” responses were grouped as “necessary” and “uncertain” and “not necessary” responses were grouped as “unnecessary”, the two groups also demonstrated different characteristics, as shown in Table 4.

**TABLE 4:** Level of decision support needs regarding HAMs (N = 966).

Variables		Necessary	Unnecessary	z	X^2^	*P*
Age [M(P25, P75), years]	—	30(26,35)	30(26,32.5)	−1.1638^#^	—	0.101
Pediatric nursing experience [M(P25, P75), years]	—	8(4,13)	7(3.5,10)	−2.539^#^	—	0.011*
HAM knowledge score less than 70 [n(%)]	Yes	208(91.2%)	20(8.8%)	—	0.261^&^	0.349
	No	681(92.3%)	57(7.7%)	—	—	—
Working department[n(%)]	ICU	310(92.3%)	26(7.7%)	—	0.038^&^	0.476
	Non-ICU	579(91.9%)	51(8.1%)	—	—	—
Primary nurse [n(%)]	Yes	645(72.6%)	244(27.4%)	—	3.107^&^	0.048*
	No	63(81.8%)	14(18.2%)	—		
Sex [n(%)]	Male	20(83.3%)	4(16.7%)	—	2.537^&^	0.117
	Female	869(92.3%)	73(7.7%)	—	—	—
With children [n(%)]	Yes	529(93.0%)	40(7.0%)	—	1.672^&^	0.121
	No	360(90.7%)	37(9.3%)	—	—	—
Bachelor’s degree or higher level of education [n(%)]	Yes	358(91.1%)	35(8.9%)	—	0.789^&^	0.398
	No	531(92.7%)	42(7.3%)	—	—	—
Intermediate or higher level of seniority [n(%)]	Yes	301(93.5%)	21(6.5%)	—	1.383^&^	0.259
	No	588(91.3%)	56(8.7%)	—	—	—
Training about HAMs [n(%)]	Yes	866(92.3%)	72(7.7%)	—	3.842^&^	0.065
	No	23(82.1%)	5(17.9%)	—	—	—

^#^Mann-Whitney *U* test, ^&^Chi square test, ^^^ *p* < 0.01, *p < 0.05.

### Preferred Forms of Decision Support About HAMs

Regarding the preferred forms of decision support for HAMs, the most popular choice was computerized clinical decision support systems (CDSSs) (448, 46.4%), and then real-time online communication with pharmacist (231, 23.9%), followed by paper books (198, 20.5%), e-books (87, 9.0%), and others (2, 0.5%).

## Discussion

This is the first study to provide data not only about the knowledge of HAM administration and management among Chinese pediatric nurses but also the basis of and confidence in decision-making and the needs for decision support about HAM administration. Our findings revealed that Chinese pediatric nurses had higher knowledge of HAMs than nurses in other countries (Kim and Kim, 2019; Zyoud et al., 2019; Salman et al., 2020). Compared to physicians in Pakistan, Chinese pediatric nurses’ knowledge of HAMs was better, with 76.4% of the nurses possessing excellent knowledge (Mustafa et al., 2022). Nevertheless, special attention should be given to the lower score for HAM regulation and the three lowest-scoring items. In fact, with the development and implementation of HAM strategies, especially information technology, including computerized prescriber order entry (CPOE), electronic medication administration records (eMAR), and barcode medication administration (BCMA), MEs in the prescription stage have been significantly reduced, making MEs in other stages of the medication process, such as preparation, distribution, administration and monitoring, an increasing proportion of all MEs (MacKay et al., 2016). Inadequate knowledge of HAMs might cause MEs, leading to poor patient outcomes. Similar to findings in Pakistan (Salman et al., 2020) and Palestine (Zyoud et al., 2019), the knowledge score of HAM regulation in Chinese pediatric nurses was significantly lower than that of HAM administration, indicating the need to improve nurses’ knowledge about HAM regulation.

The finding on the three lowest-scoring items regarding HAMs was alarming. For example, surprisingly, only one-fifth of the participants chose the right answer to the item about the fentanyl patch, which was much lower than in other countries (Zyoud et al., 2019; Salman et al., 2020; Mustafa et al., 2022). The fentanyl patch is a conventional narcotic for pain relief in adults and has several advantages, including effectiveness, good tolerance, and a long duration of action. Fentanyl-related overdose, however, has been a well-known global concern, and a recent study showed a 5.1-fold increase in fentanyl patch utilization in Australia between 2003 and 2015 (Rahman et al., 2021). Research on the pharmacokinetics and tolerability of fentanyl patches in pediatric patients has been carried out in other countries (Hiyama et al., 2021), but the patch has not yet been approved for pediatric and adolescent use in China. The U.S. Food and Drug Administration also warned of the potentially deadly exposure of fentanyl patches in children (Food and Drug Administration, 2021). In addition, 65.7% of the participants agreed with the existence of multiple concentrations to choose from for each drug, which was much higher than the percentage in previous studies (Zyoud et al., 2019; Salman et al., 2020; Mustafa et al., 2022). However, multiple concentrations and multiple dosing options may be potentially serious sources of preventable medication errors, particularly for pediatric patients receiving HAMs (Zyoud et al., 2019; Irwin et al., 2008). Early in 2004, the Joint Commission for Accreditation of Healthcare Organizations (JCAHO) in the United States recommended that hospitals stock and prepare a limited number of standard concentrations of drugs (Rich, 2004). This is especially important in pediatric acute care where errors in the calculations and preparations of individual medications in a limited time are likely to occur. Using a limited number of standard concentrations of drugs would reduce the potential for errors in solution preparation. The three lowest-scoring items provided evidence about the insufficient knowledge of nurses regarding the management of fentanyl skin patches and the error-prone use of multiple concentrations and multiple dosing regimens, indicating the need for additional continuing education and professional training on HAMs, especially for nurses who are younger, have less experience in pediatric nursing, and have a lower level of seniority.

Regarding the basis of decision-making about HAMs, our study showed that drug instructions and drug handbooks were the most frequent choices for Chinese pediatric nurses. Drug instructions should be valid, reliable, and legal texts that could guide healthcare professionals in prescription and administration and help patients to use drugs rationally. However, this is not always the case. Studies showed that there were deficiencies in the current drug instructions, including the absence of necessary content, unclear and too many nomenclatures, slow updates, and deviations from the current clinical guidelines (Chinese Clinical Medication Safety Group of International Network for the Rational Use of Drugs, 2019). More importantly, the off-label use of drugs is common in pediatrics around the world, whether in hospitals or outpatient clinics (Cui et al., 2021; Guidi et al., 2021). Therefore, drug instructions are not an ideal basis for pediatric nurses to make decisions for HAMs, let alone drug handbooks, which are potentially insufficient in terms of accuracy and timeliness. Approximately half (54.6%) of the participants considered clinical practice guidelines to be the basis of decision-making. However, clinical practice guidelines, even nationally approved guidelines, such as the United Kingdom’s Injectable Medicine Guide, might have problems with clear expression and comprehension for nurses (Jones et al., 2022). Interestingly, although the nurses’ decision-making basis about HAMs of nurses was not ideal, 84.7% of the participants reported high confidence in the decision-making about HAMs in our study, especially those who were older, having longer experience in pediatric nursing, having higher knowledge of HAMs, in position than primary nurse, having children and higher level of seniority. The improper basis of decision-making about HAM administration is extremely worrying.

To provide better support, it is essential to understand nurses’ most difficult decisions regarding HAMs. Our results showed that the most difficult decision nurses encountered when administrating HAMs was regarding the appropriateness of prescriptions. According to the Danish Patient Safety Database, Rishoej et al. (2017) described MEs in pediatric inpatients in a 5-year period, and the results showed that prescription MEs still constituted the largest part (40.8%) of all MEs. A study in China also revealed commonly inappropriate drug prescriptions in pediatric patients (Cui et al., 2021) According to the Chinese Nurses Regulation, if Chinese nurses find that a prescription violates the current laws, regulations, or standards, they should promptly report it to the responsible doctor before implementation (State Council of the People’s Republic of China, 2008). Considering the challenges faced by nurses in assessing the correctness of doctors’ prescriptions, it is understandable that nurses considered the correctness of medical orders to be the most difficult and stressful part of HAM decision-making.

In addition, the most difficult decision-making about HAMs in our study also included checks, preparation, administration, assessment, monitoring and health education, almost the entire process of medication administration. This corresponded to the high needs (92.0%) for decision support about HAMs in our results. In 2018, the American Society for Parenteral and Enteral Nutrition recommended providing clinical decision support to healthcare professionals at the time of parenteral nutrition prescription, order verification, compounding, and administration to avoid possible adverse consequences (Vanek et al., 2018). In a qualitative study about HAM administration, Sessions et al. (2019) found that current HAM safety strategies were not consistently used and recommended that education on safe HAM practices, technology enhancement, and organizational culture updates should be carried out to prevent HAM errors. In our study, the most popular types of clinical decision support were computerized CDSSs (46.4%) and real-time online communication with pharmacists (23.5%). The CDSS could facilitate evidence-based practice and greatly improve the quality of health care (Sim et al., 2001). Moss and Berner (2015) developed CDSS tools for nurses during medication administration, alerting nurses about the correct administration and monitoring of particularly dangerous aspects, such as the “Push IV dose over 4–5 min” for morphine. Such a strategy might be an opportunity to provide timely decision support for nurses during medication administration.

## Conclusion

Ensuring the medication safety of pediatric HAMs is a complex and multistep process, and pediatric nurses are in a pivotal position to prevent MEs. This study demonstrated the deficiency of pediatric nurses’ HAM knowledge, especially of HAM regulation, and the inadequate basis of and difficulty of decision-making about HAMs. Chinese pediatric nurses have a high demand for decision support about HAMs. These findings should be used to address current problems and inform possible strategies for safe practice. Nurses need more support in HAM administration, including not just training but also adequate technology, interprofessional collaboration, and organizational culture.

### Limitations

Although this study provides meaningful findings, there are still several limitations to consider. Firstly, this study was a web-based, cross-sectional study, whereby nurses were approached only once at a specific period of time. Second, the convenient sampling approach might suggest some shortcomings, such as potential sampling bias, over-representation, and non-generalizability. Moreover, the self-administered and self-reported questionnaire might have influenced the participants’ responses, as the nurses might have misjudged their competence. Furthermore, the inclusion of nurses only from tertiary hospitals may hinder the generalizability of the results to the first and secondary sectors. Studies in different settings are necessary to validate our results.

## Data Availability

The raw data supporting the conclusion of this article will be made available by the authors upon reasonable request, without undue reservation.
