# Supplementary material for: Call for Decision Support for High-Alert Medication Administration Among Pediatric Nurses: Findings From a Large, Multicenter, Cross-Sectional Survey in China
Source: Front Pharmacol. 2022 Jul 19;13:860438. doi: 10.3389/fphar.2022.860438 (PMC9343802; doi:10.3389/fphar.2022.860438)
Supplement: Supplementary file 1 [file DataSheet1.pdf]

调查问卷

亲爱的护理同仁，

您好！本问卷的调查目的是为了了解儿科护士对高警示药品的认知水平、脑力负荷、决策依据、决策信心、决策支持需求及安全文化环境情况，为后阶段构建临床决策支持系统提供实证依据。问卷不记名，大约需要花费您 10-15 分钟。

非常感谢您的支持！

（注：高警示药品，又称高危药品，本调查涉及到的高警示药品以贵院高警示药品目录为准）

第一部分　调查问卷

一、高警示药品知识问卷

下方有 20 个有关高警示药品的表述，其中前十个有关高警示药品给药方法；后十个有关高警示药品管理方法。请判断是否正确，并用√/×表示。

序号	表述	√/×
1	患者发生轻度过敏反应时，用 1：1000 肾上腺素静脉快推	
2	急救时，可用 10%$CaCl_2$10ml 在 1-2 分钟内静脉快推	
3	10%葡萄糖酸钙与 10% $CaCl_2$一样，可以互换	
4	“cc”或“ml”是胰岛素注射时使用的剂量表达	
5	计算化疗剂量时，成人以体重为基础，儿童以体表面积为基础	
6	如果发生了室颤之类的急救，可以使用 10ml 的 15%KCl 静脉快推	
7	15%KCl 最好添加到林格液中快速静滴	
8	胰岛素注射器可以用 1ml 注射器替代	
9	对于低钠患者，可以静脉快速输注 500ml 的 3%NaCl	
10	Port-A 输液泵可以用于抽血或注射药物	
11	表述剂量时，使用“安剖”或“瓶”，而非“mg”或“gm”	
12	对于外观相似药品，应使用醒目标签	
13	表述剂量时，应使用“U”而非“单位”	
14	肝素和胰岛素应一起储存在冰箱里，以便取用	
15	每个药品最好有各种浓度规格，方便护士选择	
16	如果患者可耐受，钾剂最好口服，而非静脉用药	
17	15% KCl 使用频繁，因此护士应该容易且方便获得 15% KCl	
18	对于儿童剂量，可以使用“勺”表述剂量	
19	芬太尼透皮贴剂属于常规麻醉剂	
20	如果病区里需要存放肌松剂以备气管插管，肌松剂应该与其他药物放在一起，护士可以方便获得	

二、高警示药品给药相关的临床决策信心、依据、支持需求

1. 您对自己平时<u>高警示药品给药</u>相关决策（包括并不限于核对、配置、给药方法、药效评估、不良反应的预防和监控、宣教等）的正确性，有多大的信心？

☐ 极小把握　☐ 很少把握　☐ 较小把握　☐ 较大把握　☐ 很大把握　☐ 极大把握

2. 以下是<u>高警示药品给药</u>涉及到的决策，请按照难度进行排序

☐ 核对　☐ 配置　☐ 给药方法　☐ 药效评估　☐ 不良反应的预防和监控　☐ 宣教

☐ 医嘱正确性　☐ 其他____________

3. 您平时需要进行高警示药品给药相关决策时，最主要的依据是什么？可以多选
☐ 经验　☐ 教科书　☐ 药物手册　☐ 院内规章制度
☐ 医生　☐ 药师　☐ 同事、领导　☐ 药物说明书
☐ 专题讲座　☐ 学术会议　☐ 系统评价　☐ 临床指南
☐ 原始研究　☐ 其他__________

4. 您平时高警示药品给药时是否需要决策支持？
☐ 非常需要　☐ 需要　☐ 不确定　☐ 不需要　☐ 非常不需要

5. 如果能够实现，您倾向于什么形式的高警示药品给药相关决策支持？
☐ 纸质书　☐ 电子书　☐ 药师实时交流　☐ 决策支持系统
☐ 其他______________________

第二部分　基础资料

1. 所在医院：
   ☐ 上海交通大学医学院附属上海儿童医学中心　☐ 复旦大学附属儿科医院
   ☐ 首都医科大学附属北京儿童医院　☐ 浙江大学医学院附属儿童医院
   ☐ 重庆医科大学附属儿童医院　☐ 湖南省儿童医院
   ☐ 南京医科大学附属儿童医院　☐ 广州市妇女儿童医疗中心
   ☐ 河南省儿童医院　☐ 大连市儿童医院
   ☐ 河北省儿童医院　☐ 西安市儿童医院
   ☐ 苏州儿童医院　☐ 广州孙逸仙纪念医院
2. 所在科室：
   ☐ PICU　☐ 儿童血液肿瘤科　☐ 儿童心血管科　☐ CICU　☐ 新生儿科
   ☐ NICU　☐ 小儿呼吸内科　☐ 小儿普外科
3. 性别：☐ 女　☐ 男
4. 出生年月：_______年_______月
5. 婚姻状况：☐ 未婚　☐ 已婚　☐ 离异或丧偶　☐ 其他
6. 子女状况：☐ 无　☐ 1 个　☐≥2 个
7. 第一学历：☐ 中专　☐ 大专　☐ 本科　☐ 硕士及以上
8. 现阶段学历（包括在读）：☐ 中专　☐ 大专　☐ 本科　☐ 硕士及以上
9. 职称：☐ 护士　☐ 护师　☐ 主管护师　☐ 副主任护师及以上
10. 现任角色：☐ 责任护士　☐ 协助护士
    ☐ 护理带教　☐ 护理组长　☐ 专科护士　☐ 其他_____
11. 儿科护理工作年限：_______年
12. 贵院是否有高警示药品给药相关的操作标准或实践常规？☐ 是　☐ 否
13. 近 1 年，您是否接受过高警示药品给药相关的培训？☐ 是　☐ 否
14. 近 1 年，您平时进行高警示药品给药的频率如何？
    ☐ 每天≥1 次　☐ 每周≥1 次　☐ 每月≥1 次　☐ 每年≥1 次　☐ 没有过
15. 近 1 年，您自己或您的同事是否曾经发生过给药错误事件（包括报告的、及未报告的）？
    ☐ 否　☐ 是
    给药错误事件中是否涉及到高警示药品？ ☐ 否　☐ 是

感谢您的支持！
