# Supplementary figures and images for: Call for Decision Support for High-Alert Medication Administration Among Pediatric Nurses: Findings From a Large, Multicenter, Cross-Sectional Survey in China
Source: Front Pharmacol. 2022 Jul 19;13:860438. doi: 10.3389/fphar.2022.860438 (PMC9343802; doi:10.3389/fphar.2022.860438)

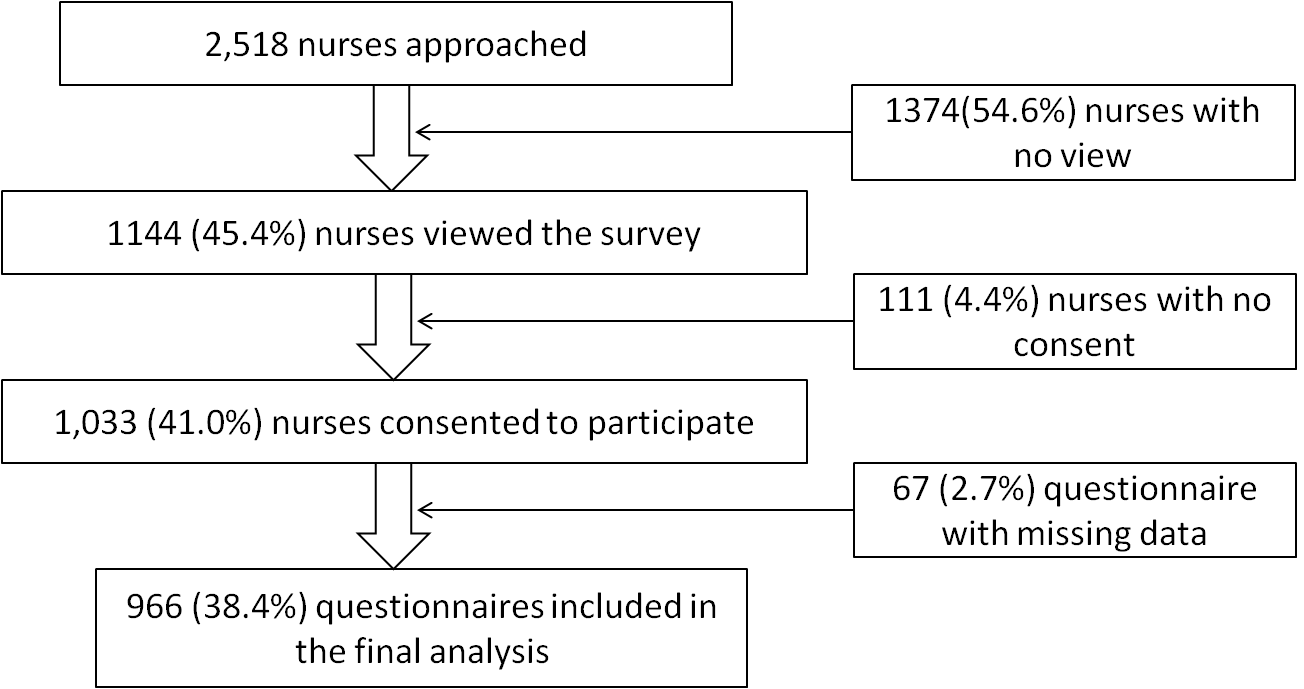

Supplement: Supplementary file 2 [file Image1.png]
